# Minimum acceptable diet and its associated factors among children aged 6–23 months in Lalibela, northeast Ethiopia: a community-based cross-sectional study

**DOI:** 10.1017/jns.2023.24

**Published:** 2023-03-31

**Authors:** Yaregal Dejene, Getachew Sale Mezgebu, Sisay Eshete Tadesse

**Affiliations:** 1 Department of Public Health, Zemen Postgraduate College, Dessie, Ethiopia; 2 School of Nutrition, Food Science and Food Technology, College of Agriculture, Hawassa University, Hawassa, Ethiopia; 3 School of Public Health, College of Medicine and Health Sciences, Wollo University, Dessie, Ethiopia

**Keywords:** Associated factor, Complementary feeding, Lalibela, Minimum acceptable diet, Minimum dietary diversity, ANC, antenatal care, AOR, adjusted odds ratio, CI, confidence interval, IYCF, Infant and Young Child Feeding, MAD, minimum acceptable diet, PCA, principal component analysis, PNC, post-natal care, sd, standard deviation, SPSS, Statistical Package for Social Sciences, WHO, World Health Organization

## Abstract

The first 2 years of life are a critical window of opportunity for ensuring optimal child growth and development. In Ethiopia, the magnitude of the minimum acceptable diet ranges from 7 to 74⋅6 %. The evidence revealed the variation and unrelated data on the prevalence of minimum acceptable diet. Therefore, the present study aimed to assess the minimum acceptable diet and its associated factors among children aged 6–23 months in Lalibela town administration, northeast Ethiopia. A community-based cross-sectional study was conducted in Lalibela town administration, northeast Ethiopia among 387 mothers/caregivers with children aged 6–23 months from May 1 to 30, 2022. The data were entered by Epidata version 3.1 and analysed by SPSS version 25.0. A multivariable binary logistic regression model was fitted to identify factors associated with minimum acceptable diet. The degrees of association were assessed using an adjusted odds ratio with a 95 % confidence interval and *P*-value of 0⋅05. The magnitude of minimum acceptable diet in the study area was 16⋅7 % (95 % confidence interval: 12⋅8–20⋅6 %). Sex of child, getting infant and young child feeding counselling at antenatal care, infant feeding practice-related knowledge and childhood illness are the variables that were found to be an independent predictor of minimum acceptable diet. Health facilities should strengthen infant feeding counselling starting from antenatal care visits during pregnancy for the recommended minimum acceptable diet is crucial.

## Background

Minimum acceptable diet (MAD) is the proportion of children aged 6–23 months who had at least minimum meal frequency and minimum diversified diet during the previous day^(1,2)^. Infants and young children (IYC) are vulnerable to malnutrition because of their high nutritional requirements for growth and development and they are particularly vulnerable during the transition period when complementary feeding begins, at 6 months^(3)^. Despite some improvements in selected nutrition indicators, progress is insufficient to meet the 2025 global nutrition targets^(4)^.

Adequate nutrition during infancy and early childhood is fundamental to the development of each child's full human potential. It is well recognised that the period from birth to 2 years of age is a ‘critical window’ for the promotion of optimal growth, health and behavioural development^(5)^.

Globally, the 2020 global nutrition report: ‘action on equity to end malnutrition’ revealed that 18⋅9 % of children aged 6–23 months received a MAD^(4)^. In African countries, Ghana, Uganda and Kenya in which 29⋅9, 23⋅9 and 48⋅5 % of children received recommended MAD, respectively^(6–8)^ and Malawi (8⋅36 %), Nigeria (7⋅3 %) and Philippines (6⋅7 %) of children aged 6–23 months received the recommended MAD^(9–13)^. In Ethiopia, the magnitude of MAD ranges from 7 to 74 %^(9,11,14–16)^.

More than two-thirds of malnutrition-related child deaths are associated with inappropriate feeding practices during the first 2 years of life^(17)^. Globally, ensuring optimal complementary feeding can avert a substantial proportion of childhood deaths^(18)^. An undernourished child has a nine times higher risk of mortality as compared with an optimally nourished child. Almost half (45 %) of all child death resulted from the effect of malnutrition, and the highest figure was in Africa^(19)^.

Previous studies conducted elsewhere on factors associated with inappropriate complementary feeding practices of children aged 6–23 months show higher maternal and paternal education, better household wealth, exposure to media, adequate antenatal and post-natal contacts, child's sex and age, institutional delivery, low parity, maternal occupation, urban residence, knowledge and frequency of complementary feeding and receiving feeding advice in immunisation as determinant factors for appropriate complementary feeding^(20–28)^.

The Ethiopian government also set targets to improve the nutritional status of children and to end child malnutrition by 2030 through implementing different programmes and strategies such as the National Nutrition Program (NNP)^(29)^, Health Sector Transformation Plan (HSTP)^(30)^, Health Extension Program (HEP)^(31)^, Sustainable Undernutrition Reduction in Ethiopia (SURE)^(32)^ and Seqota declaration^(33)^. Despite efforts done by the Ethiopian Government and other stakeholders, only 7 % of children aged 6–23 months have met the MAD^(34)^.

Even though studies were conducted about the determinants of the optimal complementary feeding practices in Ethiopia, inadequate efficient information was documented about MAD practice and its associated factors independently and most of the studies were not representative especially for rural communities^(1,14–16)^. Moreover, as far as the researcher's knowledge is concerned, no documented data were accessible specifically in the study area. Therefore, the present study aimed to assess MAD practice and its associated factors among children aged 6–23 months in Lalibela, northeast Ethiopia.

## Methods

### Study area, design and period

A community-based cross-sectional study was conducted in Lalibela town administration, northeast Ethiopia from May 1 to 30, 2022. Lalibela is found in the North Wollo zone 300 km away from Bahir Dar town which is the capital city of the region and 701 km from Addis Ababa. The town has a total population size of 42 975 people of which 21 057 are males, 21 918 are females and about 1528 estimated numbers of children 6–23 months age. There are 5 Kebeles in the town based on the Lalibela town health office 2021 report. The town has one general hospital, one health centre and six health posts.

Lalibela and its surrounding called Lasta remains in use by the Ethiopian Orthodox Christian Church to this day, and it remains an important place of pilgrimage for Ethiopian Orthodox worshipers, as a home to clergy, which increasingly brings together religious adherents. In the study area, there is household's food insecurity and their coping strategies were temporal and might not systematically link to the ever-increasing climate change and other related hazards, which were more vulnerable to food insecurity. And also, up to today, it is one site of long-term development interventions through the Government of Ethiopia-led Productive Safety Net Program (PSNP), which aims to reduce chronic food insecurity^(35)^.

### Source population and study population

All mothers/caregivers with children aged 6–23 months in Lalibela town administration were the source population. Mothers/caregivers with children aged 6–23 months who lived in the randomly selected Kebeles were the study population. Individual mothers/caregivers having children aged 6–23 months lived in the randomly selected Kebeles and participated in the actual data collection interviewee was the study unit. Mothers/caregivers with children aged 6–23 months on therapeutic feeding were excluded from the study.

### Sample size determination

The sample size was calculated for the first objective using single population proportion formula; by considering the proportion of MAD in Mareka District, southern Ethiopia 35⋅5 % of the children aged 6–23 months met the recommended MAD^(15)^, a confidence level of 95 % 1⋅96, and a 0⋅05 margin of error the sample size became 352. By adding a 10 % non-response rate, finally became 387.

### Sampling technique

There are 5 Kebeles in the Lalibela city administration, out of them 2 Kebeles (with estimated 720 study participants) were randomly selected by a lottery method. To give equal chance in the selection of mother–child pairs, a proportional allocation technique was employed across each selected Kebeles. Finally, systematic sampling techniques were applied every other lactating mother to select the study participants. If there has been more than one mother–child pair in one household unit, one mother with the youngest children is selected. In the case of twin newborns, the lottery method was used to select the study participants.

### Study variables

*Dependent variables:* Minimum acceptable diet.

*Independent variables*: Socio-demographic and economic characteristics, obstetrics and health service utilisation and maternal knowledge.

### Operational definition

- *Complementary feeding* is described as the introduction of safe and nutritionally balanced solid, semi-solid or soft foods in addition to breast milk for children aged 6–23 months^(18)^.
- *Minimum acceptable diet*: Proportion of children aged 6–23 months who had at least minimum meal frequency and minimum diversified diet during the previous day^(1,2)^.
- *Minimum meal frequency*: Proportion of breast-feeding and non-breast-feeding children aged 6–23 months who receive soft, solid and semi-solid foods (but also including milk feeds for non-breast-feed children) in the last 24 h. These food groups used for this indicator are breast milk, grains, roots and tubers; legumes and nuts; dairy products (milk, yogurt); Flesh foods (meat, fish, poultry and liver/organ meats); eggs; vitamin A-rich fruits and vegetables; and other fruits and vegetables. Quality and quantity of any amount from those groups were considered sufficient to count. Breast-feed infants aged 6–8 months two times in the last 24 h; and breast-feed infants and young children aged 9–23 months three times in the last 24 h. For non-breast-feeding infants and young children aged 6–23 months at least four times in the last 24 h^(1,36)^.
- *Minimum dietary diversity*: Proportion of children aged 6–23 months who receive five or more food groups out of the eight food groups in the last 24 h^(2)^.
- *Maternal knowledge on IYCF practice*: Mothers who score above the mean of ten knowledge questions related to infant and child feeding practice were categorised as knowledgeable and those who score below the mean were categorised as not knowledgeable^(14)^.

### Data collection tools and procedure

Data were collected by structured questionnaires using a face-to-face interview which consist the socio-demographic characteristics of mothers, history of antenatal care (ANC) visit and other variables that use to assess child-feeding practice. The questionnaire was developed by adapting from different literature^(20,22–28,37,38–41)^.

The data collectors were two nurses and two midwives who trained for 2 days regarding the purpose of the study and the procedures to be followed for data collection.

### Data quality control

The structured questionnaire was translated and prepared in Amharic and back to English by other persons to check its consistency. Two days of rigorous and extensive training were given for data collectors and supervisors on methods of obtaining consent, study objectives, contents of the questionnaire and interviewing technique prior to pre-test. A pre-test was conducted on 5 % of the total sample size on lactating women living in an area other than the study site. Overall, data collection was monitored daily and the questionnaire was checked for completeness and consistency at the end of the data collection date.

### Data processing and analysis

Data consistency and completeness were checked throughout the data collection. Data were entered using Epidata version 3.1 and exported to SPSS version 25.0 for analysis. Descriptive statistics was utilised to summarise data on respondent characteristics and presented in narrative and graphs, charts and tables. For numerical variables such as age, number of children, number of ANC mean and standard deviation were calculated for normally distributed data.

Principal component analysis (PCA) was conducted to identify variables that explained high variability among household wealth response ranked into tertile. Bartilett's test of sphericity was checked and taken *P* < 0⋅05 as significant. Sampling adequacy for principal component analysis was checked with Kaiser-Meyer-Olkin (KMO) and the measurement accepted if it was >0⋅5. Varimax rotation is employed during factor extraction to minimise cross-loading of items on many factors.

Binary logistic regression analysis was done to check the association between dependent and independent variables. All variables that had a significant association with *P*-value <0⋅25 in the bivariable analysis were selected as a candidate for multivariable logistic regression. The multivariable binary logistic regression model was fitted to identify factors affecting the MAD. Adjusted odds ratio with 95 % confidence interval and *P*-value less than 0⋅05 were considered statistically significant.

The results of multivariable logistic regression with the backward method after checking of model fitness test by the Hosmer and Lemeshow test were tested and *P*-value 0⋅196 which is greater than 0⋅05 considered as the model good fit to the data. And also, multicollinearity was checked by using a variance inflation factor: a maximum variance inflation factor (VIF) of 1⋅43 which was less than ten considered as there were no threat of multicollinearity.

### Ethical clearance

This study was conducted according to the guidelines laid down in the Declaration of Helsinki and all procedures involving human subjects/patients were approved by the ethical committee of the Zemen Postgraduate College, Department of Public Health with a reference number of ZPC/000703/2014. Written permission letter was also obtained from the Lalibela town administrative and health office. The participants enrolled in the study were informed about the study objectives, expected outcomes, benefits and the risks associated with it. Finally, verbal and written informed consent was taken and formally recorded from the participants before the interview. Furthermore, the confidentiality of participant's information was assured and information recorded anonymously. Those who are practicing inappropriate complementary feeding were advised to correct their complementary feeding practice.

## Results

### Socio-demographic and economic characteristics

A total of 359 (93 % response rate) participants were interviewed in the study period. The mean age of the infants was 13⋅31 ± 2⋅07 (sd) months and 181 (50⋅4 %) were females. More than half (54⋅3 %) of the mothers were aged between 25–34 years old and more than three-quarters (85⋅5 %) were married. More than one-thirds (39⋅6 %) mothers and 37⋅9 % of fathers attended college degree. More than two-thirds (69⋅4 %) of the respondents had a family size of ≤4 and 33⋅4 % had a poor wealth index (Table 1).

**Table 1.** Socio-demographic characteristics of study participants among children aged 6–23 months in Lalibela, northeast Ethiopia, 2022 (*N* 359)

Variable		Frequency	Percent
Age child in months	6–11	175	48⋅7
	12–17	74	20⋅6
	18–23	110	30⋅6
Sex of child	Male	181	50⋅4
	Female	178	49⋅6
Maternal age in years	25–34	95	26⋅5
	15–24	195	54⋅3
	35–49	69	19⋅2
Marital status	Married	307	85⋅5
	Divorced/separated	6	1⋅7
	Single	46	12⋅8
Maternal educational status	Unable to read and write	64	17⋅8
	Primary school (grades 1–8)	58	16⋅2
	Secondary school (grades 9–12)	95	26⋅5
	College and above	142	39⋅6
Mothers occupation	Private employee	125	34⋅8
	Governmental employee	110	30⋅6
	Merchant	42	11⋅7
	Other*	82	22⋅8
Father education status	Unable to read and write	32	8⋅9
	Primary school (grades 1–8)	72	20⋅1
	Secondary school (grades 9–12)	119	33⋅1
	College and above	136	37⋅9
Father occupation	Private employee	118	32⋅9
	Governmental employee	139	38⋅7
	Merchant	70	19⋅5
	Other*	32	8⋅9
Head of household	Husband	280	78⋅0
	Wife	79	22⋅0
Family size	≤4	249	69⋅4
	>4	110	30⋅6
Wealth index	Poor	120	33⋅4
	Medium	120	33⋅4
	Rich	119	33⋅1

* Waiter, daily labourer, unemployed.

### Obstetrics and health service utilisation

With respect to obstetrics and maternal service utilisation, the majority (76⋅6 %) of the mothers had been birth ≤2, (93⋅9 %) attended ANC during pregnancy of the index child and (59⋅6 %) received IYCF counselling during the ANC visits. Almost all mothers (90 %) attended PNC and 62⋅4 % had received IYCF counselling during their PNC visits. Almost all (92⋅2 %) were delivered in health facility and the majority (59⋅4 %) had no media access. With respect to comorbidity, 52⋅4 % had history of mother and the childhood illness (Table 2).

**Table 2.** Obstetrics and health service utilisation characteristics among children aged 6–23 months in Lalibela, northeast Ethiopia, 2022 (*N* 359)

Variable	Categories	Frequency	Percent
Birth order	≤2	275	76⋅6
	>2	84	23⋅4
ANC	Yes	337	93⋅9
	No	22	6⋅1
Received IYCF counselling during ANC	Yes	214	59⋅6
	No	145	40⋅4
PNC follow-up	Yes	323	90⋅0
	No	36	10⋅0
IYCF counselling at PNC	Yes	224	62⋅4
	No	135	36⋅4
Place of delivery	Home	28	7⋅8
	Health facility	331	92⋅2
Media access	Yes	146	40⋅7
	No	213	59⋅4
History of illness among children	Yes	189	52⋅6
	No	170	47⋅4

### Maternal knowledge and practice towards complementary feeding

**Fig. 1.**
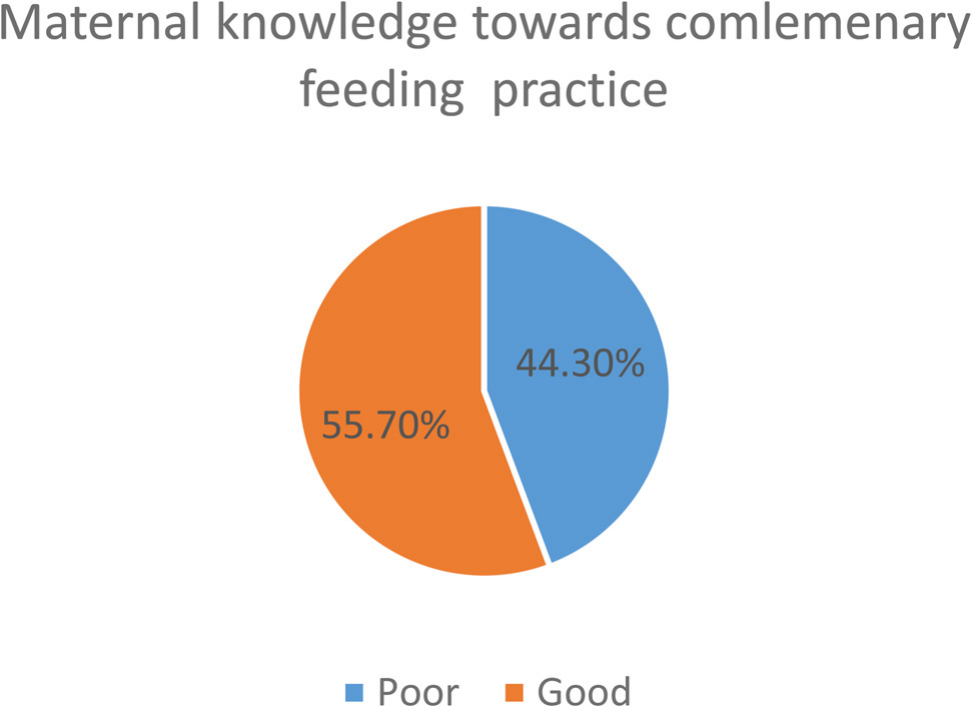
Maternal knowledge towards complementary feeding practice among children aged 6–23 months in Lalibela, northeast Ethiopia, 2022 (*N* 359).

**Fig. 2.**
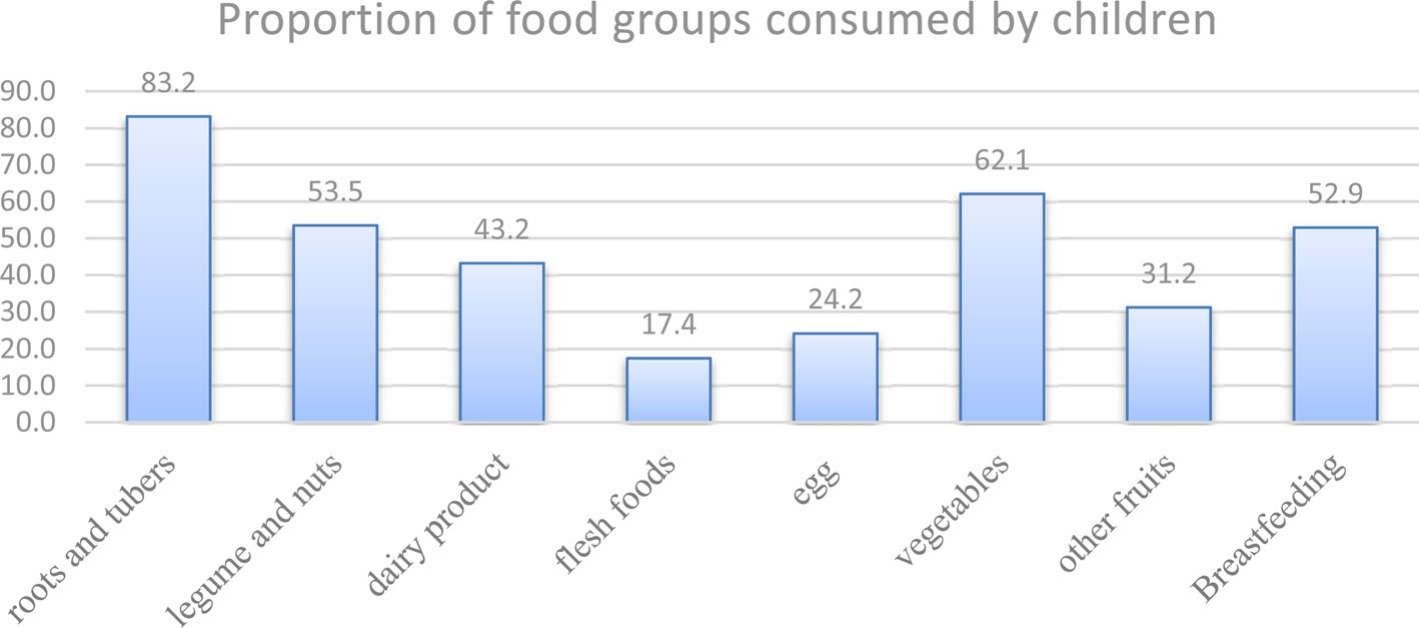
Food groups among children aged 6–23 months in Lalibela, northeast Ethiopia, 2022 (*N* 359).

**Fig. 3.**
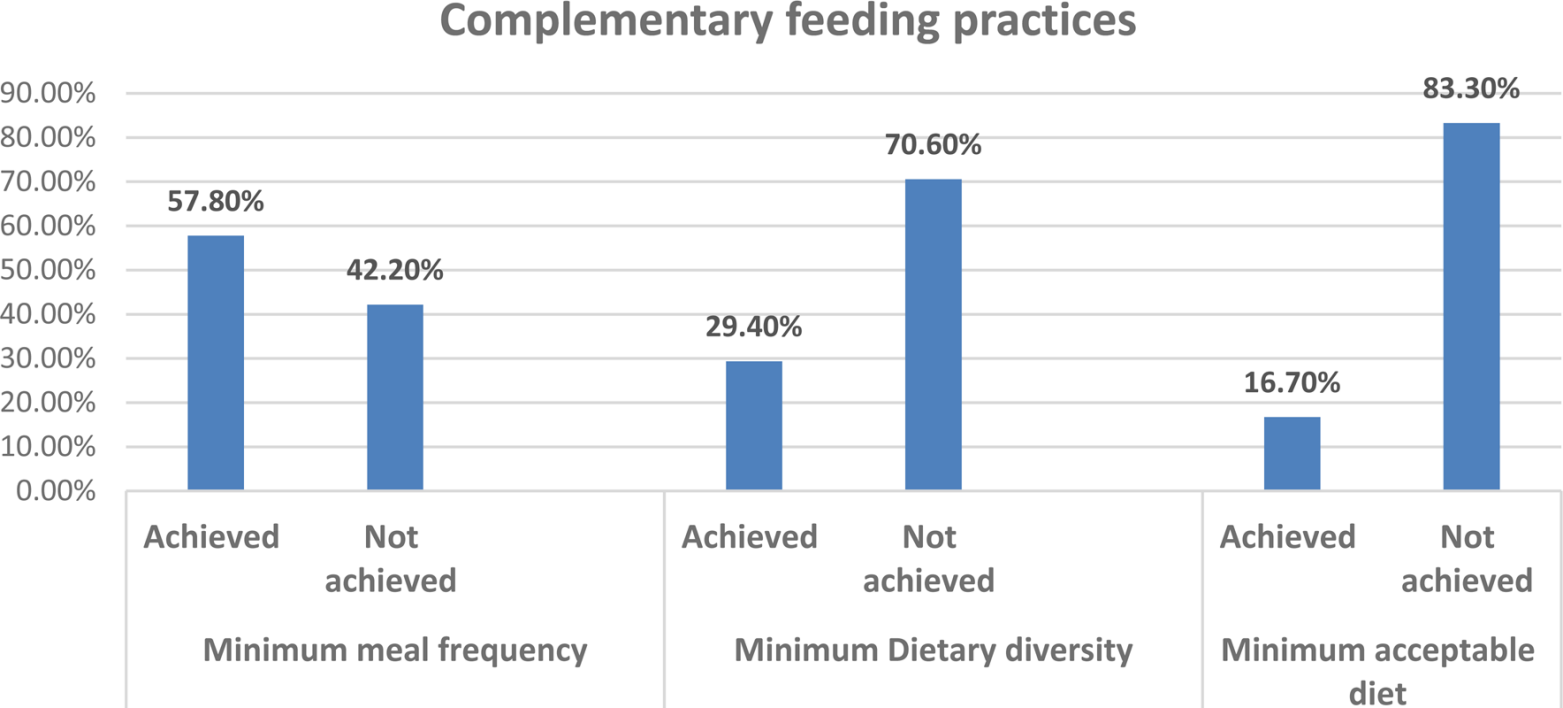
Complementary feeding practices among children aged 6–23 months in Lalibela, northeast Ethiopia, 2022 (*N*359).

**Fig. 4.**
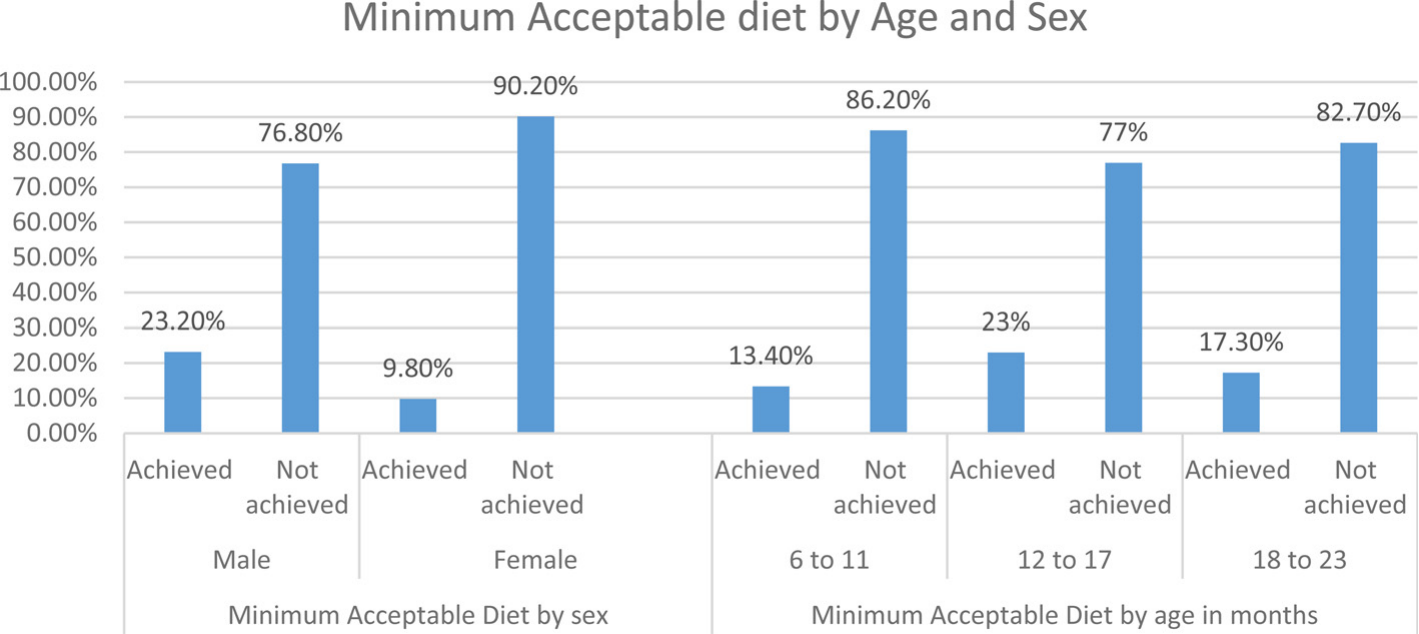
Minimum acceptable diet by age and sex among children aged 6–23 months in Lalibela, northeast Ethiopia, 2022 (*N* 359).

Nearly three-fourths (74⋅3 %) had good knowledge towards complementary feeding and half (50⋅5) of study participants ever breast-feed (Fig. 1). Grains, roots, tubers (83⋅2 %), vegetables (62⋅1 %), legumes and nuts (53⋅2 %) were the most common food items consumed by the children 24 h before the survey. But only 24⋅2 and 17⋅4 % of children consumed eggs and flesh foods, respectively (Fig. 2). About 57⋅8 % (95 % CI 52⋅3–63⋅6 %), 29⋅4 % (95 % CI 25⋅8–33⋅1 %) and 16⋅7 % (95 % CI 12⋅8–20⋅6 %) of 6–23 months aged children in Lalibela achieved minimum meal frequency, minimum dietary, diversity and minimum acceptable diet, respectively (Figs. 3 and 4).

### Factors associated with MAD

Based on the bivariable logistic regression analysis results (*P*-values <0⋅25), the variables selected for inclusion in the multiple regression model were child age, sex, maternal education level, getting IYCF counselling at ANC, place of delivery, IYCF practice counselling at PNC, infant feeding practice-related knowledge and childhood illness (Table 3).

**Table 3.** Factor associated with minimum acceptable diet among children aged 6–23 months in Lalibela, northeast Ethiopia, 2022 (*N* 359)

		MAD		COR (95 % CI)	AOR (95 % CI)	P-value
		Yes	No			
Variable		No(%)	No(%)			
Sex of child	Male	42(23⋅2)	139(76⋅8)	2⋅69(1⋅48–4⋅88)	2⋅16(1⋅04–4⋅49)	0⋅038*
	Female	18(9⋅8)	160(90⋅2)	1	1	
Child age in months	6–11	24(13⋅8)	151(86⋅2)	0⋅76(0⋅40–1⋅47)	0⋅49(0⋅18–1⋅34)	0⋅166
	12–17	17(23⋅0)	57(77⋅0)	1⋅43(0⋅69–2⋅97)	1⋅44(0⋅59–3⋅51)	0⋅421
	18–23	19(17⋅3)	91(82⋅7)	1	1	
Maternal educational status	Unable to read and write	19(29⋅7)	45(70⋅3)	2⋅19(1⋅29–4⋅39)	2⋅26(0⋅73–7⋅04)	0⋅158
	Primary school	8(13⋅8)	50(86⋅2)	0⋅83(0⋅35–1⋅98)	0⋅65(0⋅23–1⋅81)	0⋅670
	Secondary school	10(10⋅5)	85(89⋅5)	0⋅81(0⋅28–1⋅35)	0⋅35(0⋅12–1⋅04)	0⋅158
	College and Above	23(16⋅2)	119(83⋅8)	1	1	
IYCF counselling at ANC	Yes	44(20⋅6)	170(79⋅4)	1⋅91(1⋅03–3⋅54)	3⋅43(1⋅32–8⋅86)	0⋅011*
	No	16(11⋅9)	118(88⋅1)	1	1	
IYCF counselling at PNC	Yes	38(30⋅6)	186(69⋅4)	1⋅78(0⋅87–3⋅54)	1⋅18(0⋅47–2⋅99)	0⋅972
	No	11(10⋅5)	96(89⋅5)	1	1	
Place of delivery	Health facility	49(14⋅8)	282(85⋅2)	3⋅24(1⋅65–8⋅43)	1⋅03(0⋅27–4⋅16)	0⋅972
	Home	17(60⋅7)	11(39⋅7)	1	1	
Nutritional knowledge	Good	44(22⋅0)	156(78⋅0)	2⋅52(1⋅36–4⋅67)	3⋅07(1⋅41–6⋅67)	0⋅005*
	Poor	16(10⋅1)	143(89⋅9)	1	1	
Childhood illness	Yes	42(22⋅2)	147(77⋅8)	2⋅41(1⋅33–4⋅38)	5⋅87(2⋅56–13⋅45)	<0⋅001**
	No	18(10⋅6)	152(89⋅4)	1		

AOR, adjusted odds ratio; COR, crude odds ratio.

* Significant at *P*-value <0⋅05.

** Significant at *P*-value <0⋅001.

The odds of achieving MAD among male children were two times higher than females [AOR 2⋅16 (95 % CI 1⋅04–4⋅49)]. Mothers who received IYCF at ANC were 3⋅5 higher odds of minimum acceptable diet compared with mothers who had not received counselling [AOR 3⋅43 (95 % CI 1⋅32–8⋅86)]. Mothers who had good knowledge towards complementary feeding practice had three times higher odds of feeding MAD than those who have poor knowledge [AOR 3⋅07 (95 % CI 1⋅41–6⋅67)]. The odds of achieving MAD among children who were ill were six times higher than their counterparts [AOR 5⋅87 (95 % CI 2⋅56–13⋅45)].

## Discussion

To reduce malnutrition in a developing country like Ethiopia, adequate and safe infant, and young child feeding practice is crucial. The present study demonstrated that the magnitude of MAD was 16⋅7 %. Sex of child, getting IYCF counselling at ANC, infant feeding practice-related knowledge and childhood illness are the variables that were found to be an independent predictor of MAD.

This magnitude of MAD was lower compared with a study in Mareka District, southern Ethiopia 35⋅5 %^(15)^, Addis Ababa, 74⋅6 %^(16)^ and other countries like Ghana, Uganda and Kenya in which 29⋅9, 23⋅9 and 48⋅5 % of children received recommended MAD, respectively^(6–8)^ and the 2020 global nutrition report (18⋅9 %). The variation might be due to the present study was conducted in the dry season what we call ‘winter’ in which the nutritional availability of most fruits and vegetables might be low compared to seasons especially ‘summer’ a period in which this study was conducted. Moreover, the countries’ level differences might be due to differences in study design, sample size, study period and difference in socio-demographic characteristics.

In contrary, the result was higher compared with the EDHS report of 2016, only 7 % of children aged 6–23 months received a MAD^(41)^, northwest Ethiopia, Dembecha, 8⋅6 %^(9)^, rural community of Goncha District, Amhara Region, Ethiopia, 12⋅6 %^(1)^, Ethiopia multilevel analysis report of EDHS 2016 (6⋅1 %) and other countries like Malawi (8⋅36 %), Nigeria (7⋅3 %) and Philippines (6⋅7 %) of children aged 6–23 months received the recommended MAD^(9–13)^. The difference might be due to EDHS was conducted on a culturally different population, which may underrate child feeding practices while this study was conducted on an almost culturally homogeneous population with similar feeding practices. Furthermore, the reason for a high percentage of feeding practice compared to other countries might be due to variations in study design and data collection period.

The odds of achieving MAD among male children were two times higher than females. The finding was supported with the study done in Addis Ababa^(16)^, West Guji Zone, Oromia, Ethiopia^(42)^ and Sodo Zuria District^(43)^. This might be due to the cultural and/or traditional perceptions in Ethiopia mostly giving high priority to the male baby than females. This implies that female children should get attention to their requirement of dietary intake like male.

Children from mother/care who received IYCF counselling at ANC were four times more likely to achieve the MAD as compared with those who received counselling at ANC. The result was consistent with another study done in Semera logia, northern Ethiopia^(44)^ and Gondar, northwest Ethiopia^(45)^. This might be due to the fact that counselling and health education provides better health knowledge, attitude and practice towards the timely introduction of complementary feeding. This implied that ANC check-up is an appropriate time to provide essential messages about proper infant feeding practices.

Mothers who had good knowledge towards complementary feeding practices had three times higher odds of feeding MAD than those who have poor. The supporting systematic review study was found in East Africa^(46)^. This might be due to the fact that improving caregivers’ knowledge of general nutrition had a positive impact on IYCF and child health^(21)^.

The achievement of MAD among those who got sick was six times higher than the counterpart. The supporting study was found in Addis Ababa^(47)^ and Sodo town, southern Ethiopia^(48)^. This might be due to the exposure of parents to health facilities and getting health education and counselling, this leads the parent best take care of their children.

Due to the retrospective nature of the study, there might be a recall bias, especially for pregnancy-related responses like ANC visit frequency, and getting IYCF counselling at ANC. In addition, a self-reported study might not give the exact figure of the minimum dietary diversity practice (social desirability bias).

## Conclusion

The magnitude of MAD among children in the study area was unacceptably low, almost one of six children meet the recommended minimum criteria. Sex of child, getting IYCF counselling at ANC, infant feeding practice-related knowledge and childhood illness are the variables that were found to be factors associated with MAD. Health administrative should strengthen infant feeding counselling specifically about the benefit of appropriate complementary feeding and recommended MAD are important to improve the IYCF in the study area. It is important to encourage mothers for ANC and PNC check-ups to take appropriate complementary practice counselling.
